# PEI‐PLGA nanoparticles significantly enhanced the immunogenicity of IsdB_137‐361_ proteins from *Staphylococcus aureus*

**DOI:** 10.1002/iid3.928

**Published:** 2023-07-12

**Authors:** Beiyan Wang, Yazun Dong, Yuwei Cen, Shujie Chen, Xue Wen, Kaiyue Liu, Shuangshuang Wu, Liquan Yu, Yongzhong Yu, Zhanbo Zhu, Jinzhu Ma, Baifen Song, Yudong Cui

**Affiliations:** ^1^ College of Life Science and Technology Heilongjiang Bayi Agricultural University Daqing China; ^2^ Water Environmental Protection Research Institute of Daqing Oilfield Water Company Daqing China; ^3^ College of Animal Science and Veterinary Medicine Heilongjiang Bayi Agricultural University Daqing China; ^4^ Key Laboratory of Animal Epidemiology and Zoonosis, College of Veterinary Medicine China Agricul‐tural University Beijing China

**Keywords:** antibodies, bacterial, cells, infections, innate lymphocytes/Lti, molecular biology, molecules, techniques/approaches

## Abstract

**Introduction:**

*Staphylococcus aureus* seriously threatens human and animal health. IsdB_137‐361_ of the iron surface determinant B protein (IsdB) from *S. aureus* exhibits the strong immunogenicity, but its immunoprotective effect is still to be further promoted. Because PEI‐PLGA nanoparticles are generated by PEI conjugate with PLGA to develop great potential as a novel immune adjuvant, the immunogenicity of IsdB_137‐361_ is likely be strengthened by PEI‐PLGA.

**Methods:**

Here, PEI‐PLGA nanoparticles containing IsdB_137‐361_ proteins were prepared by optimizing the entrapment efficiency. Mice were immunized with IsdB_137‐361_‐PEI‐PLGA nanoparticles to assess their anti‐*S. aureus* effects. The level of IFN‐γ, IL‐4, IL‐17, and IL‐10 cytokines from spleen lymphocytes in mice and generation of the antibodies against IsdB_137‐361_ in serum was assessed by ELISA, the protective immune response was appraised by *S. aureus* challenge.

**Results:**

IsdB_137‐361_ proteins loaded by PEI‐PLGA were able to stimulate effectively the proliferation of spleen lymphocytes and increase the secretion of IFN‐γ, IL‐4, IL‐17, and IL‐10 cytokine from spleen lymphocytes, and significantly enhance generation of the antibodies against IsdB_137‐361_ in serum, reduce the level of bacterial load in liver, spleen and kidney, and greatly improve the survival rate of mice after challenge.

**Conclusion:**

These data showed that PEI‐PLGA nanoparticles can significantly enhance the immunogenicity of IsdB_137‐361_ proteins, and provide an important reference for the development of novel immune adjuvant.

## Graphical abstract

(1) PEI‐PLGA nanoparticles containing IsdB137‐361 proteins were successfully prepared by optimizi.ng the entrapment efficiency. (2) PEI‐PLGA was able to significantly enhance the immunogenicity of IsdB137‐36. (3) PEI‐PLGA is suitable for use as a novel vaccine adjuvant.

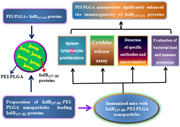

## 1 INTRODUCTION

*Staphylococcus aureus* causes multifarious infections in human and animal health, including in human‐related pneumonia, endocarditis, and mastitis in sheep and bovine.^1^, ^2^, ^3^, ^4^ Over the years, because clinical research have manifested that the vaccine is the most effective strategy in treating *S. aureus* infection, it is very necessary to develop an effective vaccine against *S. aureus*,^5^, ^6^, ^7^, ^8^ however, there is currently a lack of effective vaccine against *S. aureus* in the market. Our previous research showed that IsdB_137‐361_ of the iron surface determinant B protein (IsdB) from *S. aureus* triggers a strong immune response in animal, but its immunoprotective effect is still to be further promoted. Therefore, the need for a new adjuvant to boost the immunogenicity of IsdB_137‐361_ proteins is very important for preventing *S. aureus* infection.

Polylactic acid‐hydroxyacetic acid copolymer (PLGA) is a polymer approved by the US food and drug administration (FDA) due to its strong encapsulation and film‐forming properties, excellent biocompatibility and adjustable biodegradability,^9^ it has long been used as a scaffold or carrier for injectable drugs, vaccines, and implanting devices, so it has been widely studied. In addition, PLGA nanoparticles have a high loading capacity for insoluble drugs, and co‐delivery of antigens and adjuvants in slow‐release microparticle formulations.^10^ PLGA, as a macromolecule material with good biosafety,^11^ is hydrolyzed in vivo to produce metabolite monomers lactic acid and glycolic acid, both of which are endogenous and easily metabolized by organisms via tricarboxylic acid cycle pathway, its toxicity in drug delivery systems is minimal.^12^ Therefore, the use of PLGA nanoparticles has important implications for the design of novel drug delivery systems and medical devices.

Polyethylenimine (PEI) is a cationic polymer with linear and branched topologies and molecular weights ranging from 1 to 1000 kDa.^13^ PEI has a unique “Sponge effect,”^14^ under acidic conditions, PEI will capture a large number of protons, making the ATP‐mediated pH‐dependent proton pump open, followed by passive injection of chloride ions and water molecules, hyperosmotic state of the instantaneous outbreak, which causes the lysosomal vesicle to burst. Some of the PEI‐modified antigens could escape from lysosomes and transform into endogenous antigens presented by MHC I molecules on APC, forming antigen cross‐presentation, it greatly improves the efficiency of cell‐mediated immune response and clearance of infected cells.^15^

Iron is an important micronutrient for bacteria in the process of growth, reproduction and infection. Almost all bacterial pathogens require iron for their growth, and hemoglobin contains this metal, the role of the iron‐regulated surface determinant (Isd) system is to capture heme from hemoglobin (HB) and transport it to bacteria.^16^ The Isd system consists of four surface proteins (IsdA, B, C, H), ABC transporters with associated lipoprotein (IsdE, F), and two intracellular heme‐degrading enzymes (IsdG, I). IsdB and IsdH are unique in the Isd system because they are the only components capable of binding hemoglobin. IsdB generally promotes the adhesion and internalization of *S. aureus* to nonphagocytic human cells by binding to platelets. The results of mice immunization showed that the immunogenicity of IsdB1_37‐361_ protein was comparable to that of the whole protein. However, because the pathogenic mechanisms of *S. aureus* are very complex,^17^ and its intracellular escape strategies are perfect,^18^ the effect of immunization with IsdB_137‐361_ is not ideal, and it still needs to be further improved. In recent years, nanoparticle development provides a novel approach for drug delivery systems, particularly in PEI‐PLGA nanoparticles as pharmaceutical carriers involving wrap antigen substances such as protein, plasmid DNA and virus particles, exerting their roles to slow down the degradation and release of antigen, to promote the processing and presentation of antigen by APC, and to enhance antigen immunogenicity.^19^, ^20^, ^21^ Therefore, here, we first performed the preparation of PEI‐PLGA nanoparticles loading IsdB_137‐361_ proteins to strengthen the immunogenicity of IsdB_137‐361_.

## 2 MATERIALS AND METHODS

### 2.1 Materials

PEI‐PLGA was purchased from Jinan Daigang Bioengineering Co., Ltd. Protein Marker and BCA protein quantification kits were purchased from Solarbio Shanghai Company. Anti‐His antibody and Goat antimouse IgG‐HRP antibody were purchased from American Promega Company. His‐binding‐resin protein purification column was purchased from Shanghai Dianchuang Biotechnology Co., Ltd. IsdB_137‐361_‐pET‐28a/BL21 (DE3) and *S. aureus* Newman strain was stored in our laboratory. Specific‐pathogen‐free Kunming mice (6–8 weeks of age, female) were all purchased from Jilin Changchun Yisi Experimental Animal Technology Co., Ltd.

### 2.2 Optimization of protein entrapment efficiency by PEI‐PLGA nanoparticles

Twenty milligram PEI‐PLGA was dissolved in 1 mL of dichloromethane (DCM), and bovine serum albumin (BSA) was used as the model protein. The protein solution was prepared at a volume ratio of 1:1, 2:1, 3:1, 4:1, and 5:1 between PEI‐PLGA and BSA, respectively, and the total weight of protein m_1_ was set to 2 mg. This protein solution was treated with the ultrasonic power of 60 W in ice bath for 4 min, then the primary emulsion was added to 2% PVA solution at 1:20 drop, and the second ultrasound was performed under the same condition. Finally, the emulsion was stirred at 600 rpm/min overnight, after centrifugation of the emulsion at 4000*g* for 5 min, the supernatant was discarded to collect the microspheres, which was lysed in 1 mL of NaOH (0.1 mol/L) solution and refrigerated at 4°C overnight. On the next day, the lysate was centrifuged at 4000*g* for 10 min and the supernatant was collected. The proteins content in the supernatant were detected by BCA protein quantitative kit. The proteins packing amount m_2_ of each group of microspheres were obtained according to the standard curve of this Kit, furthermore, the protein entrapment efficiency of each group of microspheres was calculated to determine the optimal volume ratio of protein entrapped by nano‐microspheres. Encapsulation rate = m_2_/m_1_ × 100%.

### 2.3 Preparation of IsdB_137‐361_‐PEI‐PLGA nanoparticles

IsdB_137‐361_ proteins were incubated with Cy3‐labeled anti‐His antibodies of 1:1000 dilution at 37°C for 1 h. Then, IsdB_137‐361_‐PEI‐PLGA nanoparticles were prepared according to the optimal protein‐coating conditions. These nanoparticles were resuspended with ultra‐pure water and observed with fluorescence microscope. The nanoparticles were lyophilized and stored at −20°C. Some nanoparticles were put into EP tube and resuspended with aseptic deionized water, then, they were detected by scanning electronic microscopy (SEM), in addition, their properties involving size, polydispersity index (PDI) and zeta potential, was analyzed with Mastersizer. The other part of the nanoparticles were lysed with NaOH (0.1 mol/L), then, this lysate was treated and verified by SDS‐PAGE electrophoresis and Western blot.

### 2.4 Immunization of mice

The immunogens of PBS (phosphate buffer solution) + CFA, PEI‐PLGA nanoparticles, IsdB_137‐361_ proteins, IsdB_137‐361_ + CFA, and IsdB_137‐361_‐PEI‐PLGA nanoparticles were prepared, respectively, and IsdB_137‐361_ content of each group was consistent. Kunming female mice aged 6–8 weeks were randomly divided into five groups with 20 mice in each group. The prepared immunogen was injected into the legs of the mice. The protein dose of each mouse was 50 μg. After 21 days of primary immunization, the mice were inoculated with the same way.

### 2.5 Spleen lymphocyte proliferation

Seven days after the second immunization, three mice from each group were killed and their spleens were excised, the spleen lymphocytes were separated and diluted to 1 × 10^6^ cells/mL, then, the cells were cultured in a 96‐well plate with 100 μL cells per well. The IsdB_137‐361_ proteins were added in 96‐well plate at the concentration of 10 μg/mL, ConA‐stimulated cells were used as positive control, and PBS‐stimulated cells were used as negative control. After 48 h of antigen stimulation, the cell proliferation was assessed by cell counting kit (CCK)−8 according to the manufacturer's instruction. Optical Densities (OD) value was measured at 450 nm wavelength using an ELISA Reader. Stimulation index (SI) was calculated independently according to OD_450 nm_.

### 2.6 Evaluation of cytokine level

Seven days after the second immunization, the spleen lymphocytes from immunized mice were obtained and cultured in a 12‐well plate with 1 × 10^6^ cells/mL per well. The cells were treated with IsdB_137‐361_ proteins of 10 μg/mL at 37°C for 48 h, then, the cell suspensions were collected at 4°C and centrifuged at 4000*g* for 10 min, finally, the level of IFN‐γ, IL‐4, IL‐17a, and IL‐10 was detected by ELISA kit according to the instructions.

### 2.7 Bacterial load in multiple organs

Fourteen days after the second immunization, the mice were intraperitoneally injected with 5 × 10^8^ colony‐forming units (CFU) *S. aureus* strain Newman. After 48 h of challenge, three mice in each group were killed and their livers, spleens and kidneys were removed and dissected. All grinding fluids of these organs were diluted by 10, 100, and 1000 gradients, and 20 μL of each gradient was dropped into tryptic soy agar (TSA) solid plate, and cultured in 37°C incubator for 12 h, then, the colonies on the plates were counted, and the bacterial load of each organ was calculated according to the dilution ratio of the grinding solution.

### 2.8 Detection of specific antibody level

After 14 days of the second immunization, IgG antibodies were detected in sera from immunized mice by indirect ELISA. Briefly, IsdB_137‐361_ proteins were used to coat the 96‐well plates at a concentration of 10 μg/mL and incubated overnight at 4°C. After washed with phosphate buffer solution tween‐20 (PBST), the plates were blocked with 5% skim milk for 2 h at 37°C, and then the serum was diluted with PBS at 1:1000, 100 μL diluent was added into each well and incubated at 37°C for 1.5 h. After washing with PBST, HRP‐conjugated goat antimouse IgG mAbs were added at 1:5000 dilution and incubated at 37°C for 1 h. After washing, tetramethylbenzidine (TMB) solution was added and incubated with the plate for 15 min, then 2 N sulfuric acid was added to stop this reaction. Finally, antibody production was measured at OD_450 nm_ with an automated ELISA plate reader.

### 2.9 Opsonophagocytosis assay

Wild type (WT) mice were intraperitoneally injected with 1 mL (60 mg/mL) of thioglycolate solution, after 72 h, the peritoneal macrophages from the WT mice were collected and cultured in 6‐well plates at 1 × 10^6^ cells per well for 24 h. 1.5 × 10^6^ CFU of *S. aureus* strain Newman was mixed fully with 30 μL of the serum from immunized mice, and incubated at 37°C for 2 h. Then, the mixture was added into 6‐well plates according to the groups, and incubated at 37°C for 4 h, then, the gentamicin of 100 μg/mL was added into each well to remove the extracellular *S. aureus* strain Newman. After 1 h, the media in 6‐well plate was removed, and macrophages were gently washed three times with PBS, followed by the addition of cell lysate to release phagocytic bacteria. The lysates were obtained and diluted by multiple ratio. The lysates were coated on TSA plates and cultured at a 37°C for 12 h. Finally, *S. aureus* colonies were counted and the number of bacteria phagocytosed by macrophages in each group was calculated according to the dilution multiple.

### 2.10 Mice challenge

Fourteen days after the second immunization, 10 mice from each group were intraperitoneally treated with a lethal dose of 2.5 × 10^9^ CFU of *S. aureus* strain Newman. The survival number of mice in each group was counted and recorded within 14 days after challenge.

### 2.11 Statistical analysis

Experimental data were analyzed with One‐way ANOVA test using SPASS software, and final data are presented as mean ± SD, *p < .05, **p < .01, ***p < .001.

## 3 RESULTS

### 3.1 Confirmation of protein entrapment efficiency

**Figure 1:**
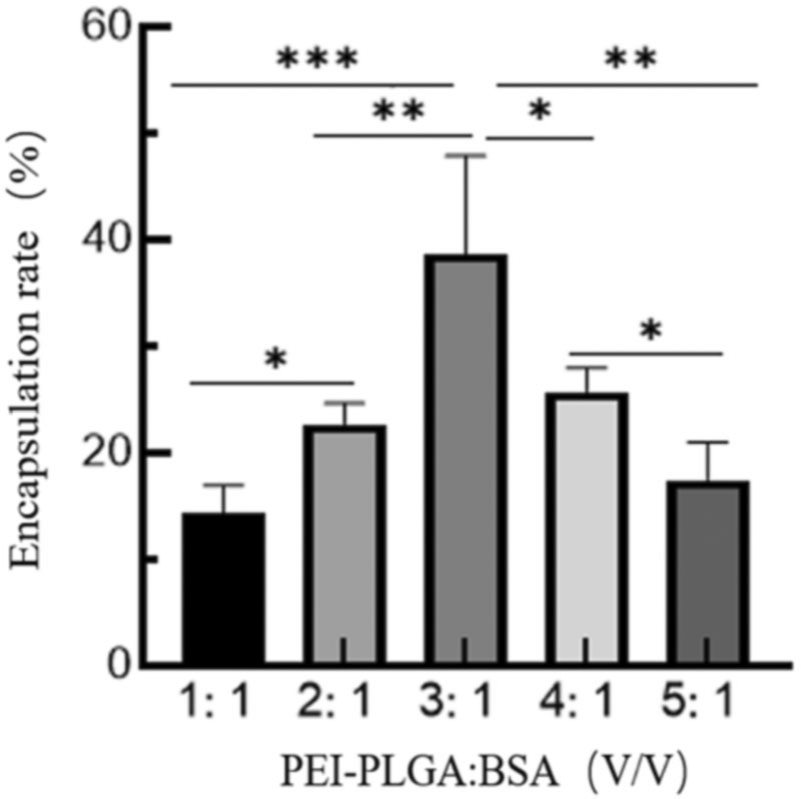
Confirmation of protein encapsulation efficiency. The optimization of protein encapsulation efficiency by PEI‐PLGA nanoparticles was confirmed according to the volume ratio of 1:1, 2:1, 3:1, 4:1, and 5:1 between PEI‐PLGA and BSA, respectively. BSA, bovine serum albumin.

The protein entrapment efficiency was confirmed according to the solution volume ratio of PEI‐PLGA and BSA. As shown in Figure 1, the entrapment efficiency of PEI‐PLGA nanoparticles first increased and then decreased with the increase of solution volume ratio. The BSA proteins were most effectively entrapped at the volume ratio of 3:1, and the entrapment efficiency of BSA protein was 40%, which was significantly higher than other volume ratio.

### 3.2 IsdB_137‐361_‐PEI‐PLGA nanoparticles were successfully prepared

**Figure 2:**
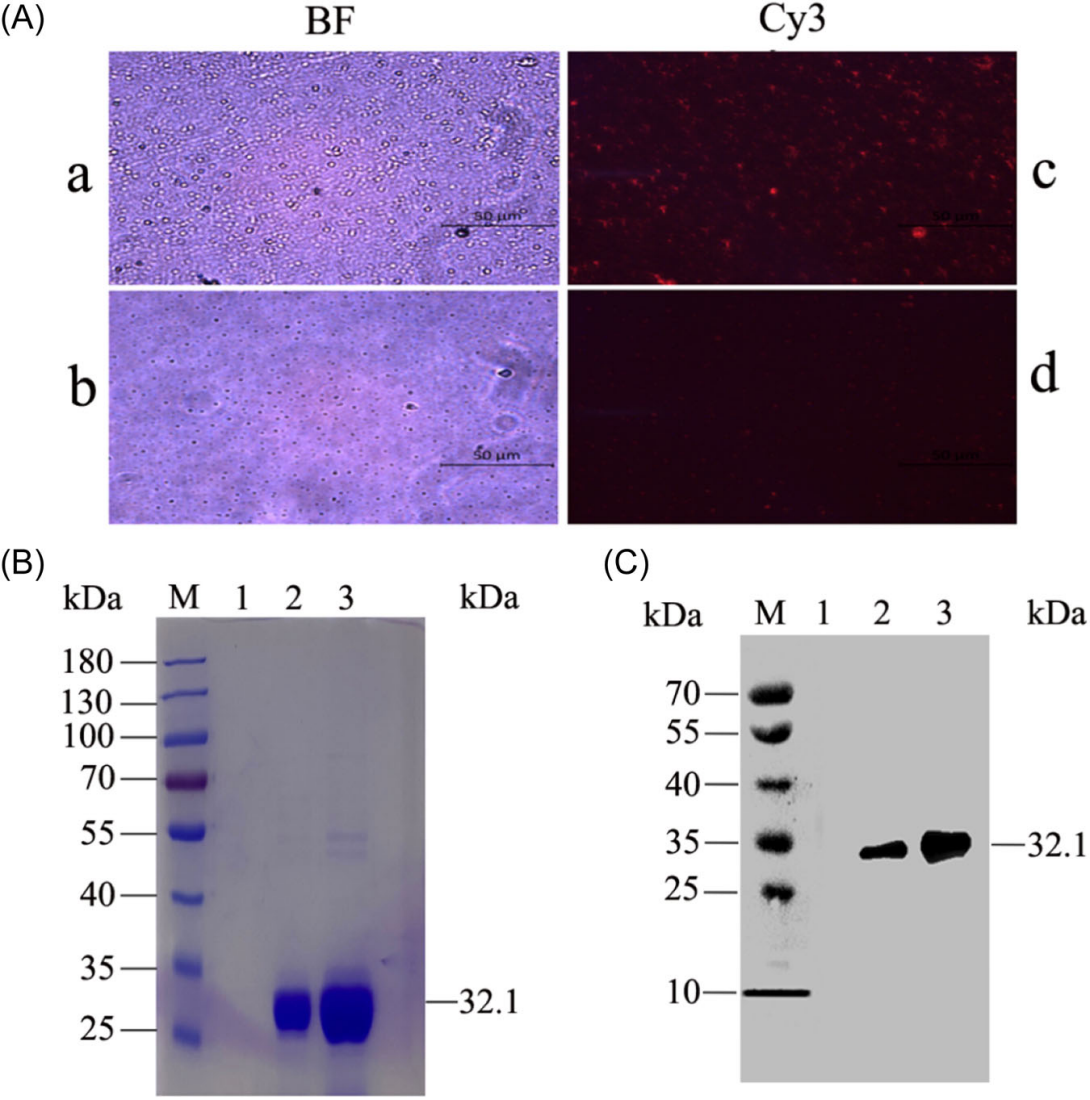
Identification of IsdB_137‐361_‐PEI‐PLGA nanoparticles. (A) Fluorescence imaging of IsdB_137‐361_‐PEI‐PLGA nanoparticles (a and b) protein‐encapsulated (a) and blank nanoparticles (b) under bright field; (c and d) protein‐encapsulated (c) and blank nanoparticles (d) under red fluorescence); (B and C) Evaluation of IsdB_137‐361_‐PEI‐PLGA nanoparticles by using SDS‐PAGE and Western blot, respectively (M: Marker; 1: Blank PEI‐PLGA nanoparticles; 2: IsdB_137‐361_‐PEI‐PLGA nanoparticles; 3: IsdB_137‐361_ proteins).

**Figure 3:**
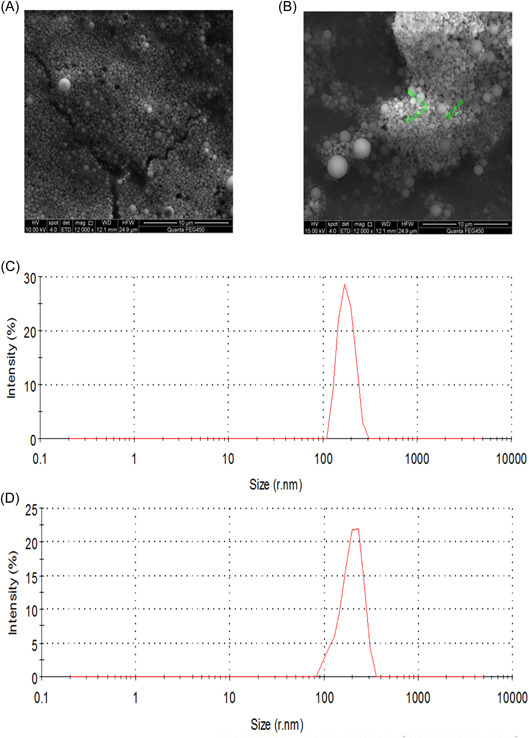
Detection of nanoparticle properties. The shape of nanoparticles was analyzed by scanning electronic microscopy. (A) Blank PEI‐PLGA nanoparticles, (B) IsdB_137‐361_‐PEI‐PLGA nanoparticles; The size of nanoparticles with Mastersizer detection. (C) Blank PEI‐PLGA nanoparticles, (D) IsdB_137‐361_‐PEI‐PLGA nanoparticles.

The IsdB_137‐361_‐PEI‐PLGA nanoparticles were prepared and observed with fluorescence microscope, as shown in Figure 2A, the PEI‐PLGA nanoparticles loading IsdB_137‐361_ proteins displayed a distinct red fluorescence, whereas the blank microspheres no fluorescence, indicating that the IsdB_137‐361_ proteins were successfully encapsulated into the nanoparticles. In addition, the IsdB_137‐361_ proteins encapsulated by PEI‐PLGA nanoparticles was identified by SDS‐PAGE and Western blot. SDS‐PAGE results revealed that the proteins of 32.1 kDa were exhibited (Figure 2B), and Western blotting analysis also showed that IsdB_137‐361_ proteins were successfully encapsulated (Figure 1C). Finally, the SEM analysis showed that the PEI‐PLGA nanoparticles and IsdB_137‐361_‐PEI‐PLGA nanoparticles presented spherical and smooth shapes (Figure 3A,B), the average size of PEI‐PLGA nanoparticles was about 250 nm (Figure 3C), with a PDI value 0.386, in contrast, the average size of IsdB_137‐361_‐PEI‐PLGA nanoparticles was about 400 nm (Figure 3D), with a PDI value 0.671, moreover, the zeta potential reading of PEI‐PLGA nanoparticles and IsdB_137‐361_‐PEI‐PLGA nanoparticles was –3.13 and –2.85 mv by Mastersizer detection, respectively. These results indicated that IsdB_137‐361_‐PEI‐PLGA nanoparticles properly exhibited the properties of nanoparticles and were successfully generated.

### 3.3 Results of spleen lymphocyte proliferation

**Figure 4:**
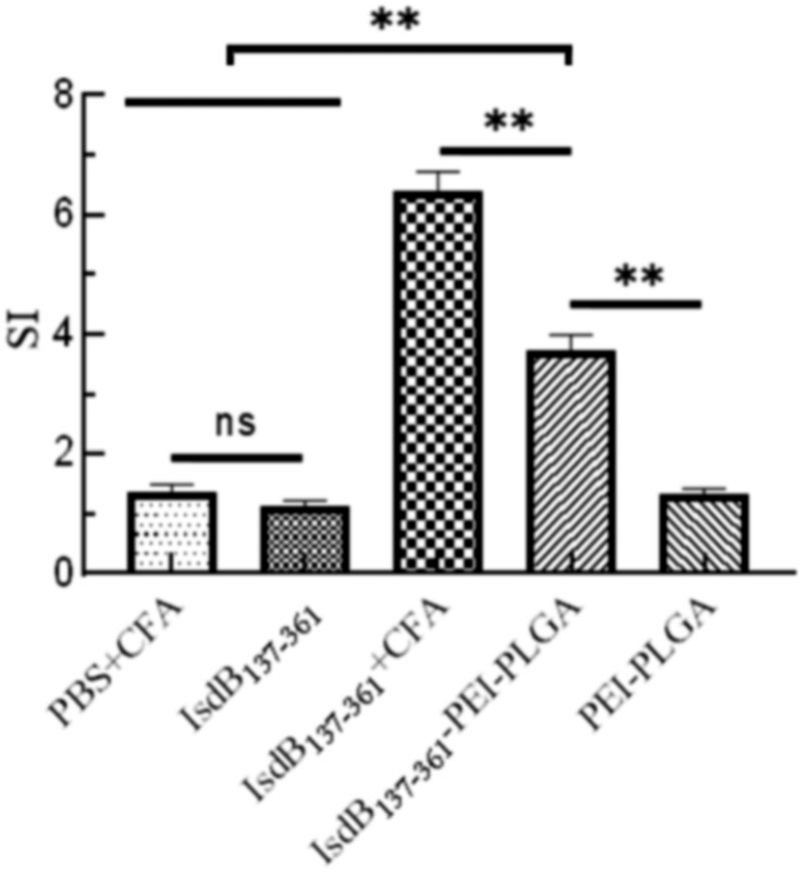
Proliferative responses of spleen lymphocyte in mice. One week after the second immunization, spleen lymphocytes were isolated from the immunized mice (*n* = 3), and spleen lymphocytes were treated with IsdB_137‐361_ proteins for 48 h. A significant difference was observed from IsdB_137‐361_‐PEI‐PLGA nanoparticles group, which was in comparison with IsdB_137‐361_ group, PBS+CFA group and PEI‐PLGA nanoparticles group. The data represent the mean ± SD (*n* = 3). Significant differences were indicated by (*p < .05), (**p < .01), and (***p < .001).

The results of splenic lymphocyte proliferation of mice in each immunization group are shown in Figure 4. The most obvious cell proliferation was observed in the IsdB_137‐361_+CFA immunization group as the positive group, which exhibited the SI of 6.5 after IsdB_137‐361_ proteins stimulation, which was much higher than that in the other immunization groups. In addition, the SI value of IsdB_137‐361_‐PEI‐PLGA nanoparticles group was 3.7, which was significantly higher than that in IsdB_137‐361_ group, PBS+CFA group and PEI‐PLGA nanoparticles group, which showed the splenocytes from the IsdB_137‐361_‐PEI‐PLGA group were significant proliferation, and indicated that PEI‐PLGA nanoparticles was able to enhance the immunogenicity of IsdB_137‐361_ proteins.

### 3.4 Detection of cytokine

**Figure 5:**
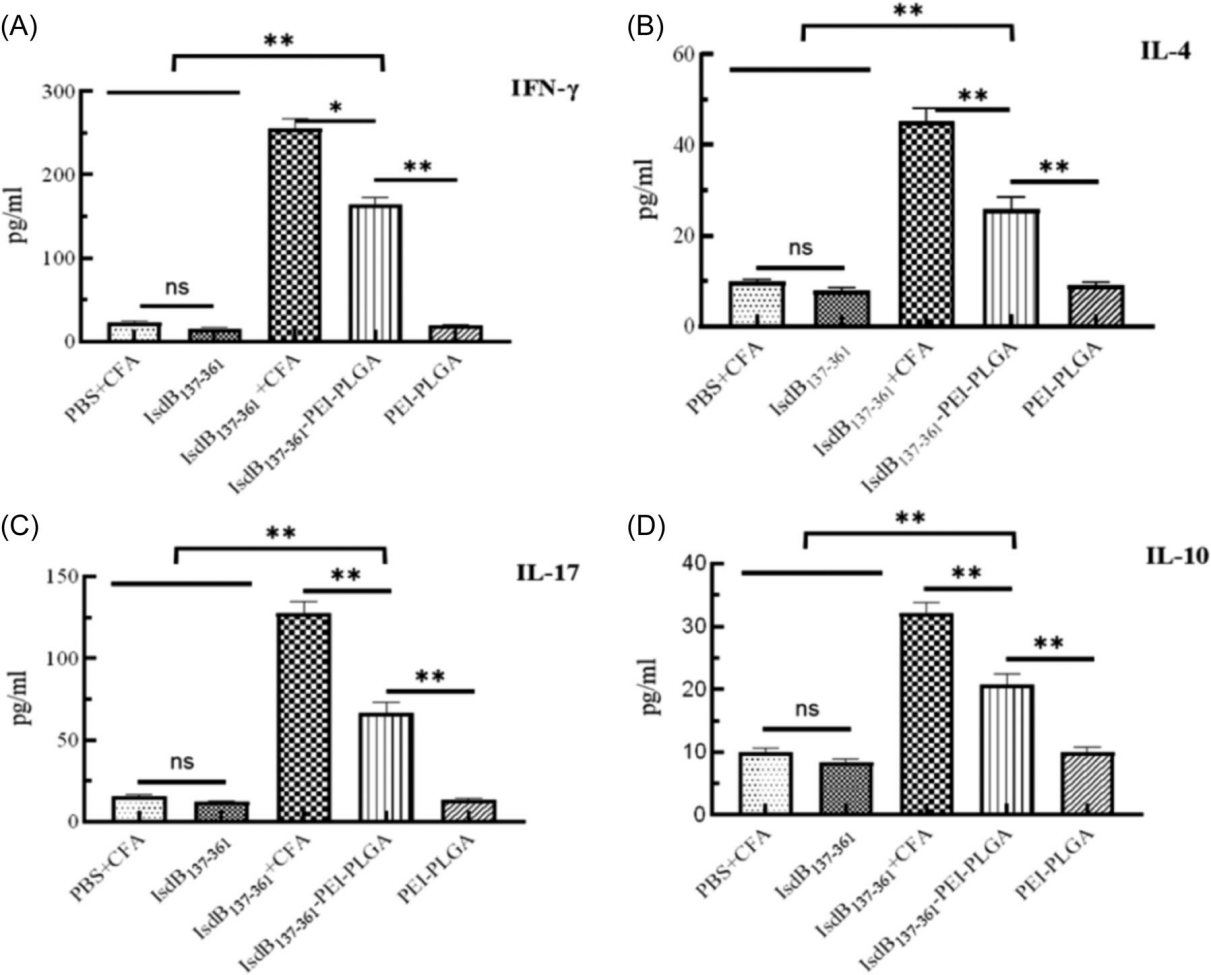
Detection of cytokine secreted by splenocytes. Splenocytes from three mice per group were isolated 2 weeks after the secondary immunization. After these splenocytes were treated with the IsdB_137‐361_ proteins, the levels of IFN‐γ, IL‐4, IL‐17, and IL‐10 were analyzed by ELISA method. The splenocyte response for IFN‐γ (A), IL‐4 (B), IL‐17 (C), and IL‐10 (D). The statistical differences between the groups are shown as **p < .01, *p < .05. The data are shown as the mean ± SD (*n* = 3).

Detection of cytokine secreted by splenocytes in each immune group was shown in Figure 5. The levels of IFN‐γ, IL‐17a, IL‐4, and IL‐10 secretion in IsdB_137‐361_+CFA group were the highest among all immune groups. However, the levels of IFN‐γ, IL‐17a, IL‐4, and IL‐10 secretion in IsdB_137‐361_‐PEI‐PLGA nanoparticles group were lower than that in IsdB_137‐361_+CFA group, but were significantly higher than that in IsdB_137‐361_ group, PBS+CFA group and PEI‐PLGA nanoparticles group, indicating IsdB_137‐361_‐PEI‐PLGA nanoparticles were able to enhance the activating of Th1, Th17, and Th2 cells.

### 3.5 Evaluation of bacterial load in organs

**Figure 6:**
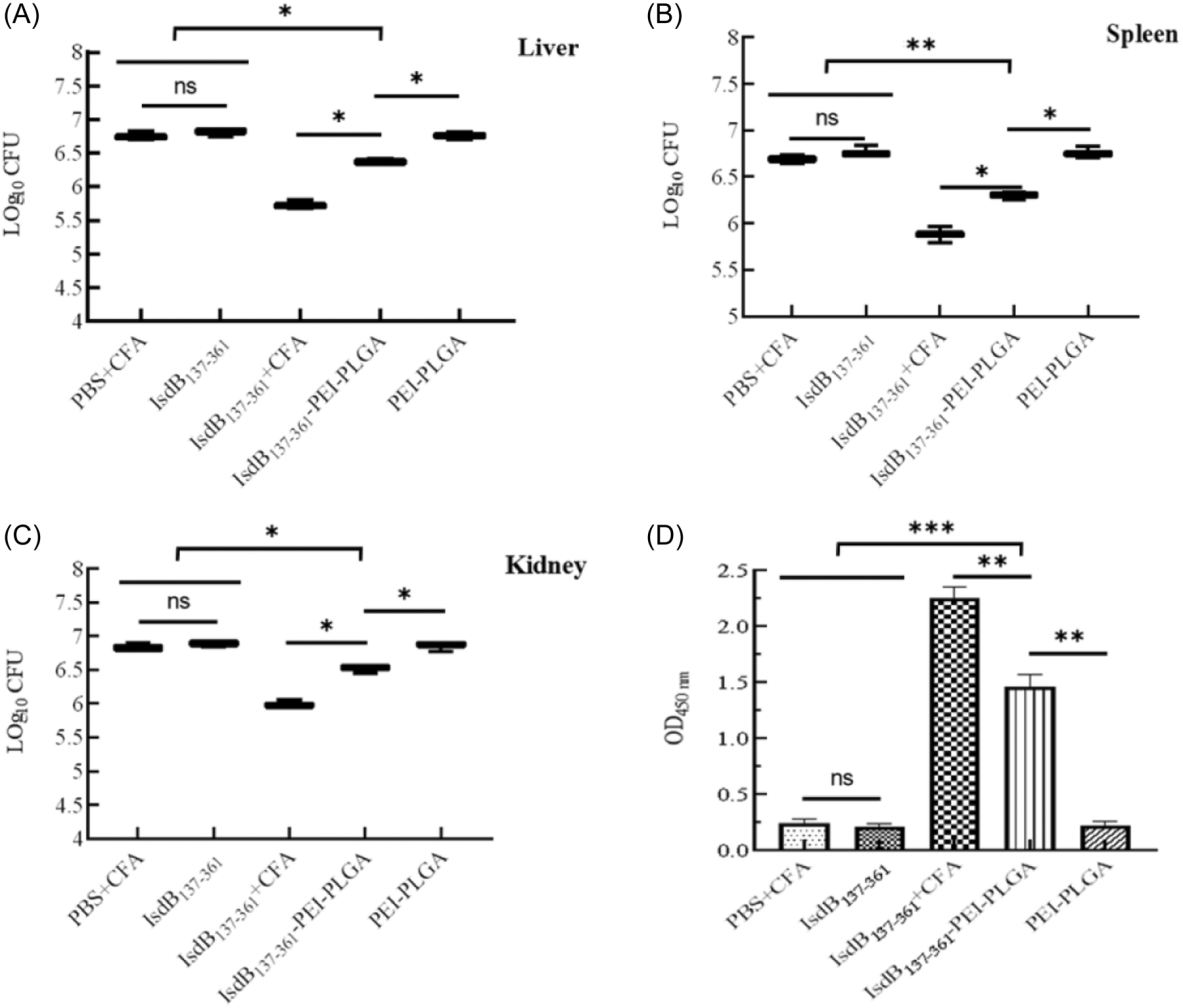
Bacterial load in organs and detection of antibody levels in mice. Fourteen days after the second immunization, the immunized mice (*n* = 3) were intraperitoneally infected with a nonlethal dose *Staphylococcus aureus* Newman for 48 h, the mice were killed, their livers (A), spleens (B), and kidneys (C) were harvested and homogenated to evaluate the CFUs of bacterial colonization. The sera in mice were analyzed by ELISA for anti‐IsdB_137‐361_ antibodies (D). The data were shown as the mean ± SD (*n* = 3). Significant differences were indicated by (*p < .05), (**p < .01), and (***p < .001). CFU, colony‐forming units.

The bacterial load in liver, spleen and kidney in each group was shown in Figure 6A–C. The bacterial load in the liver, spleen and kidney of IsdB_137‐361_+CFA group was significantly lower than that of the other groups. However, the bacterial load of IsdB_137‐361_‐PEI‐PLGA nanoparticles group was higher that of IsdB_137‐361_+CFA group. Compared with IsdB_137‐361_, PBS+CFA, and PEI‐PLGA nanoparticles groups, the bacterial load in liver, spleen and kidney of IsdB_137‐361_‐PEI‐PLGA nanoparticles group was significantly decreased, the results indicated that PEI‐PLGA nanoparticles loading IsdB_137‐361_ proteins obviously enhanced the immunogenicity of IsdB_137‐361_ proteins and could effectively protect mice against the invasive infection of *S. aureus*.

### 3.6 Detection of specific antibody levels

After the serum dilution of 1:1000 from immunized mice, the levels of IgG against IsdB_137‐361_ were detected by ELISA. As shown in Figure 6D, the mice in IsdB_137‐361_+CFA group produced the highest levels of specific antibodies, which were significantly different from other groups. The level of IgG in IsdB_137‐361_‐PEI‐PLGA nanoparticles group was also significantly higher than that of PBS+CFA group, IsdB_137‐361_ protein group and PEI‐PLGA nanoparticles group, which indicated that PEI‐PLGA nanoparticles could improve the activating B cells induced by IsdB_137‐361_ proteins and strengthen humoral immune response.

### 3.7 Results of opsonophagocytosis

**Figure 7:**
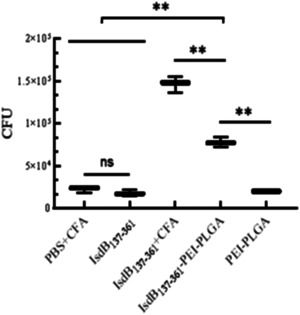
Opsonophagocytosis mediated by immune sera from mice. The sera of immunized mice were separated and mixed fully with *Staphylococcus aureus* strains Newman, and incubated at 37°C for 2 h. Then, this mixture treated mouse peritoneal macrophages at 37°C, after 4 h, the extracellular *S. aureus* Newman strains were removed by gentamicin of 100 μg/mL. Finally, these cells were lysed, and these lysates were diluted by multiple ratio. The dilute solution was cultured on TSA plates at 37°C for 12 h. The number (CFU) of bacteria phagocytosed by macrophages was calculated in each group. The data were shown as the mean ± SD (*n* = 3). Significant differences were indicated by (*p < .05) and (**p < .01). CFU, colony‐forming units.

The opsonization of serum from the immunized mice was evaluated with the opsonophagocytosis assay. As shown in Figure 7, the number of *S. aureu* phagocytosed by peritoneal macrophages of the IsdB_137‐361_+CFA group was the highest than that of the other groups. There was no significant difference among PBS+CFA, IsdB_137‐361_ protein and PEI‐PLGA nanoparticles groups, however, the number of bacteria phagocytosed by peritoneal macrophages in IsdB_137‐361_‐PEI‐PLGA nanoparticles group was significantly higher compared with the control groups except IsdB_137‐361_+CFA group. These results indicated that PEI‐PLGA nanoparticles was able to enhance the opsonization of antibodies triggered by IsdB_137‐361_ proteins in mice.

### 3.8 The survival rate of challenge

**Figure 8:**
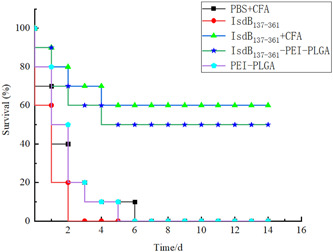
Survival rate of mice in immunized mice. There were 10 mice in each group (*n* = 10). The immunized mice were challenged with the lethal doses of *Staphylococcus aureus* strain Newman. The survival of mice was determined 14 days after challenge.

**Figure 9:**
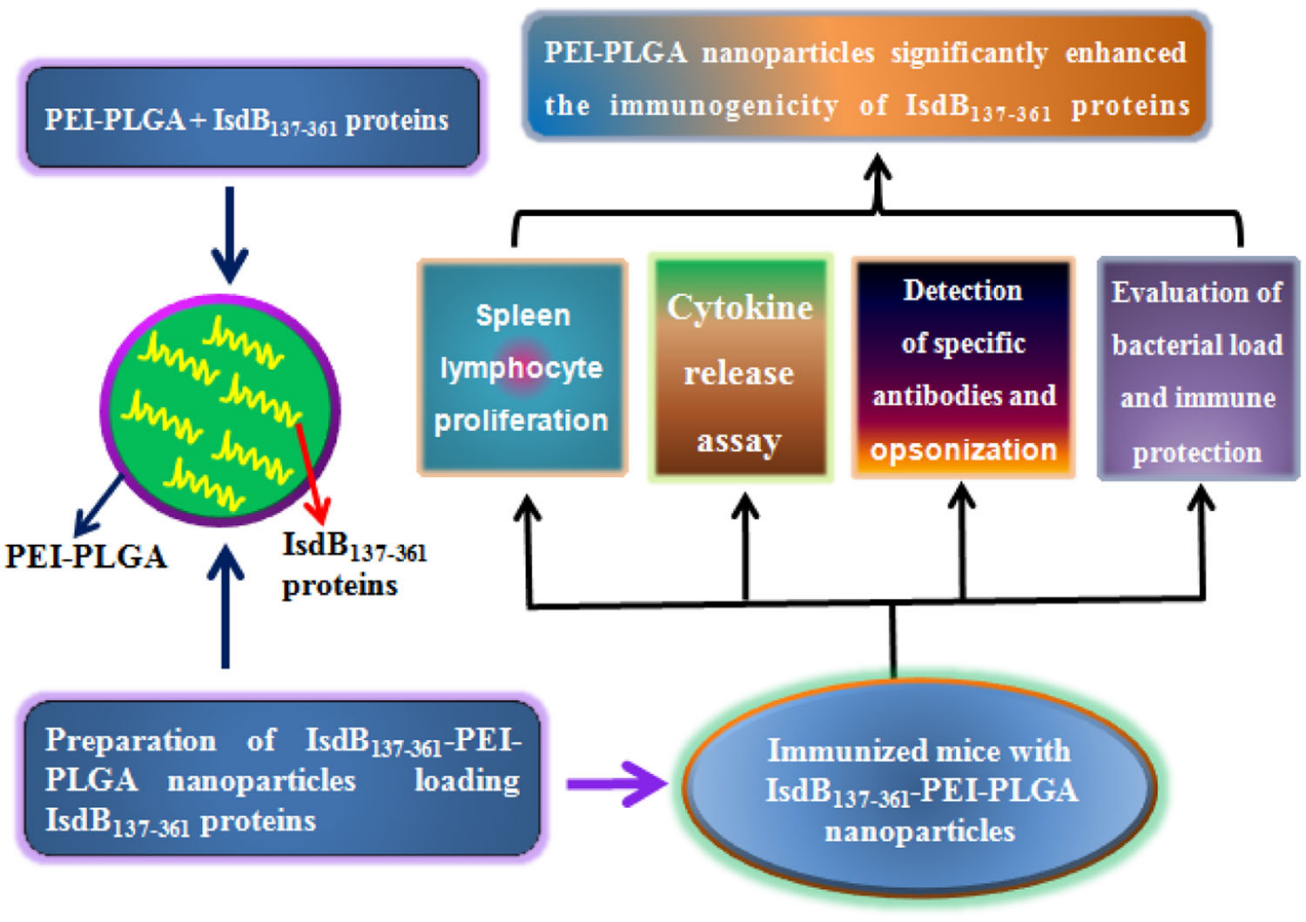
Graphical abstract. PEI‐PLGA nanoparticles containing IsdB_137‐361_ proteins were successfully generated, were able to enhance the immune responses of IsdB_137‐361_, and are suitable for use as a novel adjuvant.

The immunized mice were challenged intraperitoneally with a lethal dose of *S. aureus* strain Newman. The survival of each group within 14 days was counted to evaluate the protective efficiency. As shown in Figure 8, the IsdB_137‐361_‐immunized mice all died within 3 days of challenge, and PEI‐PLGA nanoparticles and PBS+CFA‐immunized mice also all died within 7 days of challenge. The survival rate of IsdB_137‐361_+CFA as positive control group was the highest 14 days after challenge, reaching 60%. The 14‐day survival rate of mice immunized with IsdB_137‐361_‐PEI‐PLGA nanoparticles was 50%. Compared with the negative control group, the survival rate of mice treated with IsdB_137‐361_‐PEI‐PLGA nanoparticles was significantly higher. The results indicated that PEI‐PLGA nanoparticles was able to boost the immune protective response triggered by IsdB_137‐361_ proteins in mice (Figure 9).

## 4 DISCUSSION

Most of the current vaccine antigens lack some inherent immune‐stimulating properties compared to native pathogens.^22^ Therefore, it is necessary to develop safe and effective immune adjuvant and delivery system for enhancing the immunogenicity of antigens. In the past few decades, hundreds of materials have been used as adjuvant. CFA contains an oil–water–emulsion emulsified with oil (paraffin oil or vegetable oil), emulsifier (lanolin or Twin‐80) and inactivated mycobacterium tuberculosis, CFA slows release of antigen and is easy to recruit to inflammatory‐associated cells at the injection site, favoring efficiency of antigen presentation and thus increased cell‐mediated immune response,^23^, ^24^ therefore, CFA is commonly used adjuvant in animal experiments. In addition, aluminum adjuvant, mainly including aluminum hydroxide, aluminum phosphate and alum, is able to trigger a B‐cell immune response and a CD4^+^ T‐cell immune response in the body,^25^ which is Th2‐biased in mice, but this bias is less pronounced in human immunity.^26^ The immune mechanisms of aluminum adjuvant are involved in inflammation and antigen‐presenting cell recruitment, antigen retention at the injection site, antigen uptake, T cell activation.^27^ But in some vaccines, such as typhoid and influenza vaccines, alum does not reach a satisfactory enhancement of immune response. In recent years, toll‐like receptors (TLRs) have been discovered, providing new opportunities for the development of immune adjuvants.^28^, ^29^ The TLR4 agonist monophosphoryl lipid a (MPLA) is a component in the helper system AS04 developed by GlaxoSmithKline,^30^ and MPLA is biased toward signaling with Toll/IL‐1 receptor domain‐containing adapter molecules.^31^ The cytosine preceding the guanosine (CpG) oligodeoxynucleotides (ODN) adjuvant, as short synthetic cDNA consisting of unmethylated CG dinucleotides, which binds Toll‐like receptor 9 (TLR9), and activates B cells, dendritic cells, triggers Th1‐cell‐mediated immune responses.^32^, ^33^ Despite the significant success of TLRs agonists as vaccine adjuvants, it is noteworthy that studies have displayed that, in some cases, TLRs signaling is not necessary for several standard adjuvants to induce antibody response,^34^ TLRs agonists have high potential as vaccine adjuvants, but the safety of each adjuvant‐antigen combination must be carefully assessed.^35^ However, the new adjuvants need to be found to increase the vaccine effect in future.

Nanoparticles have emerged the potential huge impact as a novel tool for vaccine developments in manipulating immune response owing to their properties.^19^, ^36^ Here, PEI‐PLGA nanoparticle was as a new adjuvant to enhance the immunogenicity of IsdB_137‐361_ proteins in mice. In the experiment of challenge, the 14‐day survival rate of IsdB_137‐361_ group was very low, and could not show the immunogenicity of the protein. The reason was probable that the challenge dose was too high, and the immune dose of protein is not high enough, in the case of not using with adjuvant, after vaccination into the body, the proteins were rapidly degraded, resulting in unsatisfactory immune effect. Although there is still a gap between the IsdB_137‐361_‐PEI‐PLGA nanoparticles and the IsdB_137‐361_+CFA for the survival rate, and lower than the IsdB_137‐361_+CFA group, but significantly higher than the IsdB_137‐361_ group and other negative controls, in addition, PEI‐PLGA nanoparticles increased obviously the immune effect triggered by IsdB_137‐361_, including the proliferation of lymphocyte, the level of cytokines, the level of specific antibodies and the bacterial load of major organs, compared with the IsdB_137‐361_ group and other negative controls, indicating PEI‐PLGA nanoparticles was a good immune adjuvant. With the deepening of the research on the application of nanoparticles, there will be more room for development of microsphere carriers. Anishbabu demonstrated in tumor cell therapy studies that the endocytosis of PLGA nanoparticles can be greatly enhanced by attaching specific target molecular receptors to the surface of PLGA nanoparticles, PLGA nanoparticles have higher cytotoxicity than PLGA nanoparticles without target modification.^37^ In 2020, Elhabak developed a long‐circulating nanocarrier consisting of Trastuzumab (TZB) surface‐modified PLGA nanoparticles co‐coated with magnolol (MAG) and gold nanoparticles (GNPs), the killing effect of the delivery system on cancer cells was tested by controlling the degree of photothermal irradiation. The results showed that the entrapment efficiency of MAG was over 81%, which improved the cytotoxicity of MAG to breast cancer cells and provided photothermal effect for multifunctional therapy of breast cancer cells. The encapsulated photothermal material GNPs enables the nano‐carrier to give near‐infrared photothermal response, which improves the cytotoxicity of drugs and enhances the specificity to cancer cells.^38^ We thus hypothesized that targeting antigen present cells modification of IsdB_137‐361_‐PEI‐PLGA nanoparticles might have a better immune effect, but this needs to be verified by subsequent experiments. However, an adjuvant often triggers a weak immune response against antigens, the combined adjuvants act synergistic effect to maximize the immunogenicity of antigens. Hence, we predicted that PEI‐PLGA nanoparticles combines with other adjuvants, mainly including MPLA, CPG, and aluminum adjuvants, could generate a synergistic effect to heighten the immune response against antigens, which would be responsible for driving the action of B cells and Th1, Th2, Th17 cells. In this study, *S. aureus* strain Newman only was used to perform for the challenge experiment, to further evaluate the immunogenicity of IsdB_137‐361_‐PEI‐PLGA nanoparticles, other *S. aureus* strains, including *S. aureus* strain Wood46 and *S. aureus* strain Heilongjiang23‐1, will be selected to finish challenge experiment in the future.

In conclusion, IsdB_137‐361_‐PEI‐PLGA nanoparticles obviously stimulated the proliferation of spleen lymphocytes and improved the generation of IFN‐γ, IL‐4, IL‐17, and IL‐10 cytokines, significantly increased the levels of the antibodies against IsdB_137‐361_ in mice, decreased the bacterial load in liver, spleen and kidney, and greatly enhanced the immune protective effect in mice. Taken together, our results verified that PEI‐PLGA nanoparticles were able to enhance the immunogenicity of IsdB_137‐361_ proteins, which provides an important reference for the development of novel adjuvant and *S. aureus* vaccine.

## AUTHOR CONTRIBUTIONS

Beiyan Wang contributed design of the study, drafted, and revised the article. Yazun Dong performed the experiments and analyzed the data. Yuwei Cen, Shujie Chen, Xue Wen, Kaiyue Liu, Shuangshuang Wu, Liquan Yu, Yongzhong Yu, Zhanbo Zhu, Jinzhu Ma, Baifen Song, and Yudong Cui contributed to data collection.

## CONFLICT OF INTEREST STATEMENT

The authors declare no conflict of interest.

## ETHICS STATEMENT

This study was performed in accordance with the recommendations in the Guide for the Care and Use of Laboratory Animals of Heilongjiang Bayi Agricultural University. The experimental protocols were approved by the Institutional Animal Care and Use Committee at Heilongjiang Bayi Agricultural University.

## Data Availability

The data that support the findings of this study are available.

## References

[1] Li Q , Huang KX , Pan S , Su C , Bi J , Lu X . Thymol disrupts cell homeostasis and inhibits the growth of *Staphylococcus aureus* . Contrast Media Mol Imaging. 2022;2022:8743096. 36034206 10.1155/2022/8743096 PMC9392601

[2] Galar A , Weil AA , Dudzinski DM , Muñoz P , Siedner MJ . Methicillin‐resistant *Staphylococcus aureus* prosthetic valve endocarditis: pathophysiology, epidemiology, clinical presentation, diagnosis, and management. Clin Microbiol Rev. 2019;32:1‐26. 10.1128/CMR.00041-18 PMC6431130 30760474

[3] Azara E , Foddai AC , Longheu CM , Addis MF , Tola S . Production of recombinant proteins including the B‐cell epitopes of autolysin A of *Staphylococcus aureus* isolated from clinical sheep mastitis and their potential for vaccine development. Vet Res Commun. 2023:1‐10. 10.1007/s11259-023-10121-1 PMC10113713 37074614

[4] Gharaibeh MH , Abu‐Qatouseh LF . First molecular characterization of capsule expression and antibiotic susceptibility profile of *Staphylococcus aureus* isolates from bovine mastitis in Jordan. Vet World. 2022;15:2269‐2274. 36341060 10.14202/vetworld.2022.2269-2274 PMC9631364

[5] Miller LS , Fowler VG , Shukla SK , Rose WE , Proctor RA . Development of a vaccine against *Staphylococcus aureus* invasive infections: evidence based on human immunity, genetics and bacterial evasion mechanisms. FEMS Microbiol Rev. 2020;44:123‐153. 31841134 10.1093/femsre/fuz030 PMC7053580

[6] Proctor RA . Is there a future for a *Staphylococcus aureus* vaccine? Vaccine. 2012;30:2921‐2927. 22115633 10.1016/j.vaccine.2011.11.006

[7] Schaffer AC , Lee JC . Staphylococcal vaccines and immunotherapies. Infect Dis Clin North Am. 2009;23:153‐171. 19135920 10.1016/j.idc.2008.10.005

[8] Chand U , Priyambada P , Kushawaha PK . *Staphylococcus aureus* vaccine strategy: promise and challenges. Microbiol Res. 2023;271:127362. 36958134 10.1016/j.micres.2023.127362

[9] Lü J‐M , Wang X , Marin‐Muller C , et al. Current advances in research and clinical applications of PLGA‐based nanotechnology. Expert Rev Mol Diagn. 2009;9:325‐341. 19435455 10.1586/erm.09.15 PMC2701163

[10] Essa D , Kondiah PP , Choonara YE , Pillay V . The design of poly (lactide‐co‐glycolide) nanocarriers for medical applications. Front Bioeng Biotechnol. 2020;8:48. 32117928 10.3389/fbioe.2020.00048 PMC7026499

[11] Li G , Zhao M , Xu F , et al. Synthesis and biological application of polylactic acid. Molecules. 2020;25:5023. 33138232 10.3390/molecules25215023 PMC7662581

[12] Kasturi SP , Skountzou I , Albrecht RA , et al. Programming the magnitude and persistence of antibody responses with innate immunity. Nature. 2011;470:543‐547. 21350488 10.1038/nature09737 PMC3057367

[13] Neu M , Fischer D , Kissel T . Recent advances in rational gene transfer vector design based on poly (ethylene imine) and its derivatives. J Gene Med. 2005;7:992‐1009. 15920783 10.1002/jgm.773

[14] Akinc A , Thomas M , Klibanov AM , Langer R . Exploring polyethylenimine‐mediated DNA transfection and the proton sponge hypothesis. J Gene Med. 2005;7:657‐663. 15543529 10.1002/jgm.696

[15] Al‐Deen FN , Selomulya C , Ma C , Coppel RL . Superparamagnetic nanoparticle delivery of DNA vaccine. Methods Mol Biol. 2014;1143:181‐194. 24715289 10.1007/978-1-4939-0410-5_12

[16] Pishchany G , Sheldon JR , Dickson CF , et al. IsdB‐dependent hemoglobin binding is required for acquisition of heme by *Staphylococcus aureus* . J Infect Dis. 2014;209:1764‐1772. 24338348 10.1093/infdis/jit817 PMC4038968

[17] Kwiecinski JM , Horswill AR . *Staphylococcus aureus* bloodstream infections: pathogenesis and regulatory mechanisms. Curr Opin Microbiol. 2020;53:51‐60. 32172183 10.1016/j.mib.2020.02.005 PMC7244392

[18] Huitema L , Phillips T , Alexeev V , Tomic‐Canic M , Pastar I , Igoucheva O . Intracellular escape strategies of *Staphylococcus aureus* in persistent cutaneous infections. Exp Dermatol. 2021;30:1428‐1439. 33179358 10.1111/exd.14235 PMC8110615

[19] Al‐Jubori AA , Sulaiman GM , Tawfeeq AT , Mohammed HA , Khan RA , Mohammed SAA . Layer‐by‐layer nanoparticles of tamoxifen and resveratrol for dual drug delivery system and potential triple‐negative breast cancer treatment. Pharmaceutics. 2021;13:1098. 34371789 10.3390/pharmaceutics13071098 PMC8309206

[20] Mahinfar P , Mokhtarzadeh A , Baradaran B , Siasi Torbati E . Antiproliferative activity of CD44 siRNA‐PEI‐PEG nanoparticles in glioblastoma: involvement of AKT signaling. Res Pharm Sci. 2022;17:78‐85. 34909046 10.4103/1735-5362.329928 PMC8621842

[21] Jwameer MR , Salman SA , Noori FTM , et al. Antiproliferative activity of PEG‐PEI‐SWCNTs against AMJ13 breast cancer cells. J Nanomater. 2023;2023:1‐8.

[22] Harandi AM , Davies G , Olesen OF . Vaccine adjuvants: scientific challenges and strategic initiatives. Expert Rev Vaccines. 2009;8:293‐298. 19249971 10.1586/14760584.8.3.293

[23] Hawksworth D . Advancing Freund's and addaVax adjuvant regimens using CpG oligodeoxynucleotides. Monoclon Antib Immunodiagn Immunother. 2018;37:195‐199. 30281392 10.1089/mab.2018.0022

[24] Dubé J‐Y , McIntosh F , Zarruk JG , David S , Nigou J , Behr MA . Synthetic mycobacterial molecular patterns partially complete Freund's adjuvant. Sci Rep. 2020;10:5874. 32246076 10.1038/s41598-020-62543-5 PMC7125112

[25] Burny W , Callegaro A , Bechtold V , et al. Different adjuvants induce common innate pathways that are associated with enhanced adaptive responses against a model antigen in humans. Front Immunol. 2017;8:943. 28855902 10.3389/fimmu.2017.00943 PMC5557780

[26] Zhou C , Li C , Gong GZ , et al. Analysis of immunological mechanisms exerted by HBsAg‐HBIG therapeutic vaccine combined with Adefovir in chronic hepatitis B patients. Hum Vaccines Immunother. 2017;13:1989‐1996. 10.1080/21645515.2017.1335840 PMC5612521 28665747

[27] Hem SL , Hogenesch H . Relationship between physical and chemical properties of aluminum‐containing adjuvants and immunopotentiation. Expert Rev Vaccines. 2007;6:685‐698. 17931150 10.1586/14760584.6.5.685

[28] Anthoney N , Foldi I , Hidalgo A . Toll and Toll‐like receptor signalling in development. Development. 2018;145:1‐6. 10.1242/dev.156018 29695493

[29] Kumagai Y , Takeuchi O , Akira S . Pathogen recognition by innate receptors. J Infect Chemother. 2008;14:86‐92. 18622669 10.1007/s10156-008-0596-1

[30] Garçon N , Chomez P , Van Mechelen M . GlaxoSmithKline Adjuvant Systems in vaccines: concepts, achievements and perspectives. Expert Rev Vaccines. 2007;6:723‐739. 17931153 10.1586/14760584.6.5.723

[31] Mata‐Haro V , Cekic C , Martin M , Chilton PM , Casella CR , Mitchell TC . The vaccine adjuvant monophosphoryl lipid A as a TRIF‐biased agonist of TLR4. Science. 2007;316:1628‐1632. 17569868 10.1126/science.1138963

[32] Kayraklioglu N , Horuluoglu B , Klinman DM . CpG oligonucleotides as vaccine adjuvants. Methods Mol Biol. 2021;2197:51‐85. 32827132 10.1007/978-1-0716-0872-2_4

[33] Simon R , Diaz‐Rosales P , Morel E , Martin D , et al. CpG oligodeoxynucleotides modulate innate and adaptive functions of IgM(+) B cells in rainbow trout. Front Immunol. 2019;10:584. 30972075 10.3389/fimmu.2019.00584 PMC6443966

[34] Gavin AL , Hoebe K , Duong B , et al. Adjuvant‐enhanced antibody responses in the absence of toll‐like receptor signaling. Science. 2006;314:1936‐1938. 17185603 10.1126/science.1135299 PMC1868398

[35] Zeng Z , Wang H , Zhang Z , Yi Y . Research progress of new vaccine adjuvants. Shengwu Gongcheng Xueba = Chin J Biotechnol. 2021;37:78‐87. 10.13345/j.cjb.200220 33501791

[36] Zhao L , Seth A , Wibowo N , et al. Nanoparticle vaccines. Vaccine. 2014;32:327‐337. 24295808 10.1016/j.vaccine.2013.11.069

[37] Babu A , Amreddy N , Muralidharan R , et al. Chemodrug delivery using integrin‐targeted PLGA‐Chitosan nanoparticle for lung cancer therapy. Sci Rep. 2017;7:14674. 29116098 10.1038/s41598-017-15012-5 PMC5676784

[38] Elhabak M , Osman R , Mohamed M , El‐Borady OM , Awad GAS , Mortada N . Near IR responsive targeted integrated lipid polymer nanoconstruct for enhanced magnolol cytotoxicity in breast cancer. Sci Rep. 2020;10:8771. 32472087 10.1038/s41598-020-65521-z PMC7260181

